# Personalized, Web-Based, Guided Self-Help for Patients With Medically Unexplained Symptoms in Primary Care: Protocol for a Randomized Controlled Trial

**DOI:** 10.2196/13738

**Published:** 2019-10-08

**Authors:** Anne van Gils, Denise Hanssen, Antoinette van Asselt, Huibert Burger, Judith Rosmalen

**Affiliations:** 1 Interdisciplinary Center Psychopathology and Emotion Regulation University Medical Center Groningen University of Groningen Groningen Netherlands; 2 Department of Epidemiology University Medical Center Groningen University of Groningen Groningen Netherlands; 3 Department of General Practice University Medical Center Groningen University of Groningen, Groningen Netherlands

**Keywords:** medically unexplained symptoms, somatoform disorders, precision medicine, eHealth, general practice

## Abstract

**Background:**

Medically unexplained symptoms (MUS) constitute a major health problem because of their high prevalence, the suffering and disability they cause, and the associated medical costs. Web-based interventions may provide an accessible and convenient tool for managing MUS. We developed a personalized, Web-based, guided self-help intervention for MUS in primary care (Grip self-help) and would compare its effectiveness with that of usual care.

**Objective:**

This paper aims to describe the rationale, objectives, and design of a pragmatic randomized controlled trial (RCT) assessing the effectiveness of Grip self-help.

**Methods:**

For a pragmatic multicenter RCT, 165 adult patients with mild to moderate MUS will be recruited through general practices in the Netherlands. Randomization will be performed at general practice level. Over the course of several months, patients in the intervention group will receive a personalized set of Web-based self-help exercises, targeting the unhelpful cognitions, emotions, behaviors, and social factors that are relevant to them. The intervention is guided by a general practice mental health worker. The control group will receive care-as-usual. Primary outcome is physical health-related quality of life (RAND-36 or 36-item general health survey, physical component score). Secondary outcomes include severity of physical and psychological symptoms, mental health–related quality of life, cost-effectiveness, and acceptability. Assessments will take place at baseline, end of treatment, and at 16-, 26-, and 52-week follow-ups.

**Results:**

Recruitment started in December 2018, and enrolment is ongoing. The first results are expected to be submitted for publication in December 2021.

**Conclusions:**

To our knowledge, this is the first study to combine the concepts of electronic health, self-help, and personalized medicine in the treatment of MUS. By improving the quality of life and reducing symptoms of patients with MUS, Grip self-help has the potential to reduce costs and conserve scarce health care resources.

**Trial Registration:**

Dutch Trial Register NTR7390; https://www.trialregister.nl/trial/7390.

**International Registered Report Identifier (IRRID):**

PRR1-10.2196/13738

## Introduction

### Background

In primary care, about 50% of patients presenting with a physical complaint receive a medical diagnosis during their first visit. After extensive evaluation, approximately one-third of physical symptoms remain medically unexplained [1]. Medically unexplained symptoms (MUS) can cause significant distress and impairment for patients and are associated with high costs for society because of the resulting excess use of health care services, work absenteeism, and decreased productivity [2-4].

Although the pathophysiology of MUS is unknown, a lot has been published on factors that might trigger and maintain symptoms [5,6]. Targeting these factors, such as worries, fear, and physical inactivity, is the focus of most psychological treatments. Cognitive behavioral therapy is well studied and has shown modest improvements with regard to symptom severity and physical health–related quality of life (HRQoL) [7,8]. However, most patients with MUS are treated in primary care, and general practitioners (GPs) generally lack the time and skills to offer psychological treatment. More in general, GPs often find it difficult to treat patients with MUS [9] because of the lack of available and effective treatment options [8,10].

A recent meta-analysis has shown that self-help interventions are a promising alternative to psychological treatment for patients with MUS [11]. As self-help does not require guidance by a trained therapist, it can be easily accessible and widely available at relatively low costs, especially when offered on the Web.

### Grip Self-Help

We, therefore, developed the Web-based intervention *Grip self-help*. Grip self-help is a personalized, guided self-help intervention for patients with mild to moderate MUS in primary care. On the basis of the results of Web-based questionnaires, patients receive a personalized set of Web-based self-help exercises, aimed at the unhelpful cognitions, emotions, behaviors, and social factors that are relevant to them. The intervention has an eclectic nature and contains elements of patient education, cognitive behavioral therapy, acceptance and commitment therapy, and problem-solving treatment. As far as we know, no previous research evaluated the effectiveness of such an intervention.

### Objective

This paper describes the design of the randomized controlled trial (RCT) assessing the effectiveness of the Grip self-help intervention in general practice (Dutch Trial Register NTR7598). The primary objective of this RCT is to determine whether Grip self-help is superior to care-as-usual (CAU) for improving physical HRQoL at follow-up after 16 weeks in patients with mild to moderate MUS. Secondary objectives are as follows: to (1) assess the effectiveness of Grip self-help in comparison with CAU in improving severity of physical and psychological symptoms and mental HRQoL at follow-up after 16, 26, and 52 weeks, (2) investigate the cost-effectiveness of Grip self-help in comparison with CAU at follow-up after 16, 26, and 52 weeks, (3) assess acceptability of Grip self-help for patients and primary care professionals (PCPs), (4) investigate which patient characteristics predict effectiveness of Grip self-help, (5) investigate which characteristics of PCPs predict effectiveness of Grip self-help, and (6) investigate whether increased self-efficacy mediates treatment outcomes.

## Methods

### Study Design

This study is designed as a pragmatic multicenter randomized controlled superiority trial with 2 parallel groups and a 1:1 allocation ratio. The study protocol, intervention, participant information, and informed consent procedure have been approved by the University Medical Center Groningen Medical Ethics Committee (registration number M18.232173). The study will be conducted according to the principles of the Declaration of Helsinki (2013 version).

### Participants

**Figure 1:**
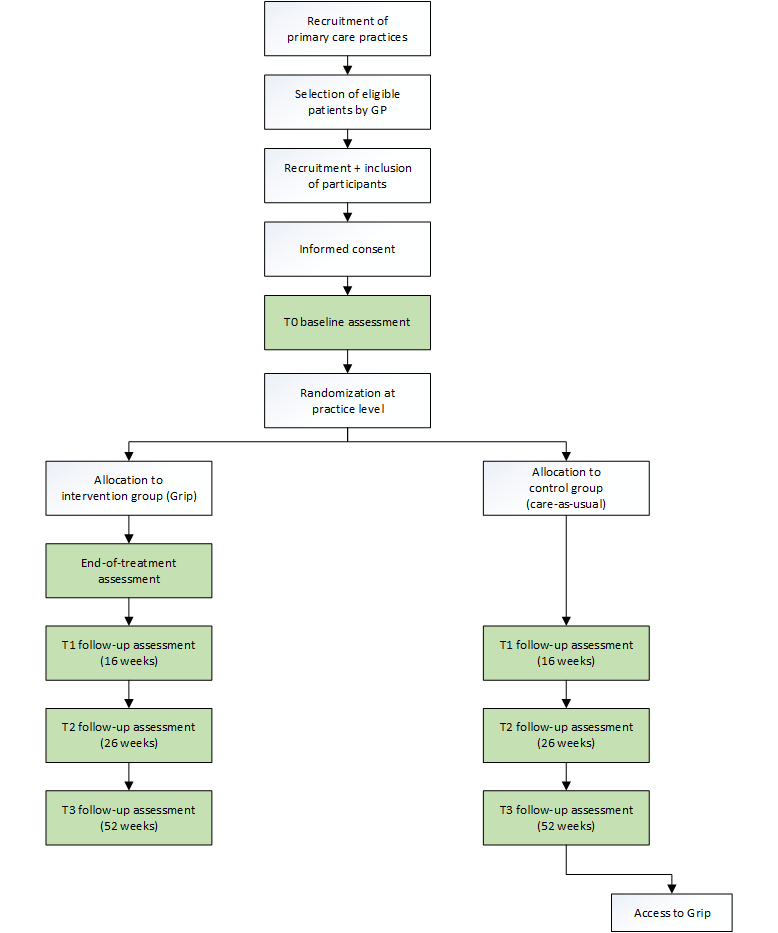
Flowchart of the study procedure. GP: general practitioner.

Patients with mild to moderate MUS will be recruited through general practices from rural as well as urban areas in the Netherlands. Of the PCPs, 2 types will be involved in this study: GPs and general practice mental health workers (GP-MHWs). GP-MHWs are nurses, psychologists, or social workers with experience in mental health care, employed by one or several general practices. PCPs will be invited to participate through local and national GP networks, social media, and Web publicity. PCPs that show interest will be informed by a letter. If desired, more detailed information can be provided by email, telephone, or during a visit to the practice. At least 1 GP and 1 GP-MHW are required to participate for a practice to take part in the study. Participating PCPs sign an informed consent form. Subsequently, the GP selects up to 15 patients with mild to moderate MUS based on the inclusion criteria described in Textbox 1. Selected patients receive a letter with information about the study. During a telephone call with one of the researchers, additional questions from interested patients will be answered and exclusion criteria (see Textbox 1) will be evaluated. When eligible patients decide to participate in the study, they will be asked to sign an informed consent form. Next, the participant will receive an email with an invitation to fill out the baseline questionnaires in a Web-based secure environment. An overview of the study procedure is provided in Figure 1.

#### Eligibility Criteria for Participating General Practices

*Inclusion criteria:* At least 1 GP and 1 GP-MHW from the practice take part in the study.

#### Eligibility Criteria for Participants

Textbox 1 presents the inclusion and exclusion criteria for participants.

**Inclusion and exclusion criteria for participants.**

Inclusion criteria

- Age ≥18 years
- Presenting with mild to moderate medically unexplained symptoms (MUS). In line with the guidelines provided by the Dutch College of General Practitioners, MUS are defined as “physical symptoms that have persisted for more than several weeks and for which adequate medical examination has not revealed any condition that sufficiently explains the symptoms” [12]. MUS are considered mild to moderate in case of (1) mild to moderate functional limitations because of the symptoms, (2) symptoms in 1, 2 (mild), or 3 (moderate) symptom clusters (gastrointestinal symptoms, cardiopulmonary symptoms, musculoskeletal symptoms, and nonspecific symptoms), and (3) symptom duration longer than expected by the general practitioner
- Main symptom concerns pain, gastrointestinal complaints, or fatigue
- Adequate command of the Dutch language, no major cognitive or visual impairment

Exclusion criteria

- Referred to or currently treated by a mental health professional
- Start or adjusted dosage of psychotropic medication ≤3 months ago
- Likelihood of posttraumatic stress disorder (Trauma Screening Questionnaire ≥6 [13]), severe anxiety disorder (4-Dimensional Symptom Questionnaire or 4DSQ Anxiety ≥10 [14]), or severe depressive disorder (4DSQ Depression ≥6 [14])
- Pregnancy
- Engaged in a legal procedure concerning disability-related financial benefits
- Not in possession of an email account and a personal computer, laptop, or tablet with internet connection

### Randomization Procedure

Randomization will be performed at general practice level. After all participants from a practice have given informed consent and filled out baseline questionnaires, practices will be randomly assigned to the intervention (Grip self-help) or control (CAU) group, using Web-based randomization tool ALEA (ALEA Clinical | FormsVision). Randomization after patient inclusion prevents the possibility of recruitment bias (selection bias). Randomizing general practices rather than patients will avoid PCPs within a practice offering both Grip self-help and CAU, as this could cause contamination effects. Randomization will take place in blocks, randomly varying in size between 4 and 8, and a 1:1 allocation ratio.

### Control Group

Participants assigned to the control group will receive CAU during the study period. This could include care by the GP, GP-MHW, physiotherapist, and a psychologist. After the last follow-up measurement at 52 weeks, participants assigned to the control group will be offered access to the study intervention.

### Intervention Group

**Figure 2:**
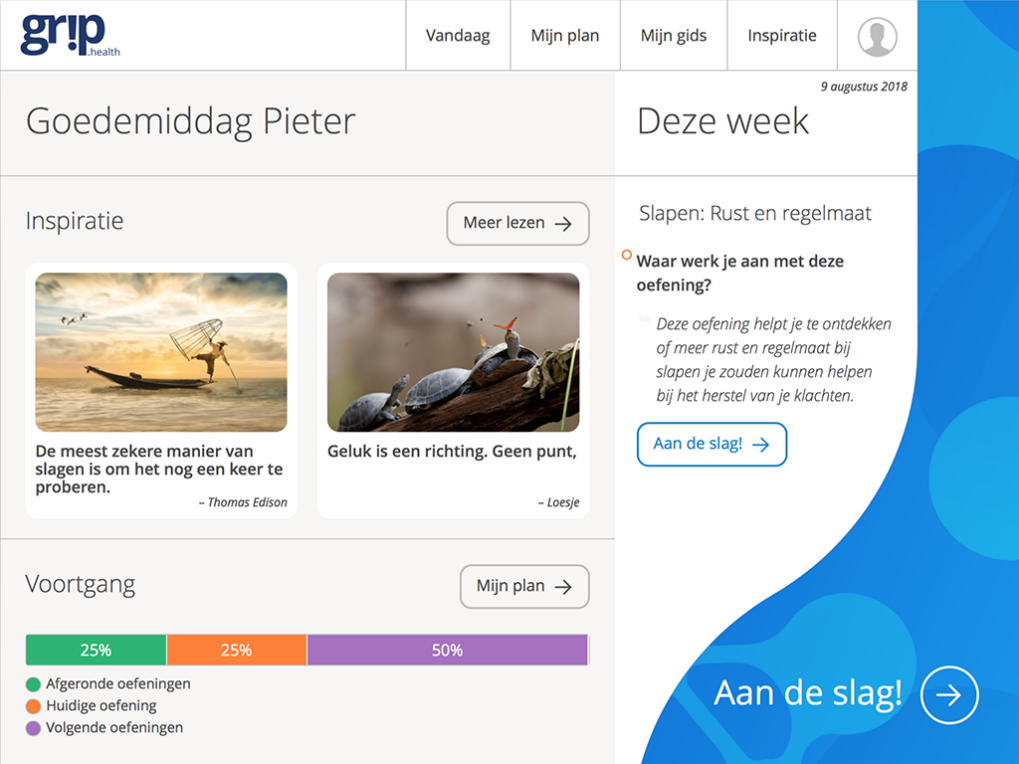
Screenshot of the homepage of the patient interface of Grip self-help.

In addition to CAU, participants in the intervention group will be offered a Web-based self-help intervention called *Grip self-help*. Figure 2 shows a screenshot of the patient interface of the intervention.

The intervention comprises 2 steps. First, participants fill out a set of Web-based questionnaires concerning potential perpetuating factors: unhelpful cognitions, emotions, behaviors, and social factors associated with the physical symptoms. With this information, a personal problem profile is generated, identifying perpetuating factors that are relevant to the individual. Second, participants gain access to Web-based self-help exercises, selected using personalization algorithms based on their problem profile. Exercises are selected from a database, containing 59 unique exercises. Exercises include education, adjusting life style, identifying and challenging unhelpful cognitions, relaxation and mindfulness exercises, learning to accept the presence of physical symptoms and negative emotions, identifying values and setting goals accordingly, gradual exposure to feared activities, and managing the impact of symptoms on work and relationships. The exercises were not written from the perspective of a single therapeutic theoretical framework. Rather, they contain elements of cognitive behavioral therapy, acceptance and commitment therapy, and problem-solving treatment. The exercises vary with regard to duration (1 or 2 weeks) and intensity (varying from a single assignment to daily practice). Patients will work on 1 exercise at a time. The intervention will ultimately result in a personalized self-help guide, composed of texts that are extracted from the exercises patients found useful during the intervention.

The intervention is guided by the GP-MHW. A Web-based manual and technical support via email will be available to GPs and GP-MHWs allocated to the intervention group. These GPs and GP-MHWs will also be offered the option to take a free Web-based course on MUS and working with Grip.

GP-MHWs will be instructed to invite patients for at least two visits (start and finish). The frequency of further visits is left up to the GP-MHW. Although the exact length of the intervention will vary per person, we estimate that participating in the Grip self-help intervention on average takes 16 weeks. In 16 weeks, the participant will complete approximately 6 to 8 exercises.

### Outcomes and Assessments

Outcome measures at the patient level will be assessed at baseline, end of treatment, and follow-up after 16, 26, and 52 weeks. Physical HRQoL at 16 weeks, measured with the physical component score of the RAND-36, will be the primary outcome measure. Physical HRQoL at 26 and 52 weeks will be secondary outcome measures, as well as mental HRQoL, symptom severity (physical and psychological symptoms), and costs (health care utilization and productivity loss) after 16, 26, and 52 weeks. In addition, patient satisfaction with the study intervention will be assessed at 16 weeks and at the end of treatment. As the duration of the intervention will vary among participants, completion of the last self-help exercise is considered *end of treatment*. An overview of the assessment schedule can be found in Tables 1 and 2.

**Table 1:** Patient questionnaires and assessment schedule.

Questionnaire	Variable	T^a^0	End of treatment^b^	T1 (16 weeks)	T2 (26 weeks)	T3 (52 weeks)
Demographics	Age, sex, education, marital status	X	—^c^	—	—	—
RAND-36^d^	Physical and mental health–related quality of life	X	X	X	X	X
4DSQ^e^	Symptom severity physical and psychological symptoms	X	—	X	X	X
*i*MCQ^f^	Health care utilization	X	—	X	X	X
*i*PCQ^g^	Productivity loss	X	—	X	X	X
SCQ-8^h^	Patient satisfaction	—	X	X	—	—
SES^i^	Self-efficacy	X	X	X	—	—

^a^T: time point.

^b^End of treatment: after the last self-help exercise has been completed, these questionnaires are only filled out by participants in the intervention group.

^c^Not applicable.

^d^RAND-36: 36-item general health survey.

^e^4DSQ: 4-Dimensional Symptom Questionnaire.

^f^*i*MCQ: Medical Consumption Questionnaire.

^g^*i*PCQ: Productivity Costs Questionnaire.

^h^SCQ-8: Social Communication Questionnaire–8.

^i^SES: Self-Efficacy scale.

**Table 2:** Health care professional questionnaires and assessment schedule.

Questionnaire	Variable	T^a^0	End of treatment^b^	T1 (16 weeks)	T2 (26 weeks)	T3 (52 weeks)
MUS^c^ attitude questionnaire	Attitude toward MUS	X	—^d^	—	—	—
DIBQ^e^	Determinants of implementation behavior	X	—	—	—	—
Electronic health attitude questionnaire	Attitude toward electronic health	X	—	—	—	—
SCQ-3^f^	Health care provider satisfaction	—	X	—	—	—

^a^T: time point.

^b^End of treatment: after the last patient has completed the last self-help exercise; these questionnaires are only filled out by health care professionals in the intervention group.

^c^MUS: medically unexplained symptoms.

^d^Not applicable.

^e^DIBQ: Determinants of Implementation Behavior Questionnaire.

^f^SCQ-3: Social Communication Questionnaire–3.

All instruments are self-report questionnaires. Participants will receive automated emails containing a link to the questionnaires. If participants have not filled out the questionnaires, automated email reminders will be sent after 1 and 2 weeks. If participants have not filled out the questionnaires after these reminders, a research assistant will call to remind them.

If participants decide to withdraw from the study before they have completed the study protocol, the main reason for withdrawal will be inquired. Also, participants will be asked to complete the Web-based questionnaires at follow-up after 16, 26, and 52 weeks.

### Instruments

#### Health-Related Quality of Life

We will use the validated Dutch version of the 36-item General Health Survey (RAND-36) to assess HRQoL. The RAND-36, which is nearly identical to the SF-36, is a self-report questionnaire for measuring general health status [15,16]. In this study, the 8 subscales will be aggregated into 2 summary scores: the physical and mental component score. The physical component score comprises 4 subscales: general health, bodily pain, physical functioning, and role limitations because of physical problems. The mental component score also comprises 4 subscales: vitality, mental health, social functioning, and role limitations because of emotional problems. Scores range between 0 and 100, with a higher score representing a better HRQoL*.*

#### Symptom Severity

Severity of MUS will be assessed with the Somatization subscale of the 4-Dimensional Symptom Questionnaire (4DSQ). The 4DSQ is a validated 50-item Dutch self-report questionnaire, developed and widely used in general practice to assess somatization, distress, anxiety, and depression [14]. The somatization subscale considers the frequency of 16 common physical symptoms over the past week with a score range between 0 and 32.

The 4DSQ will also be used to assess distress (subscale with 16 items, score range 0-32) and symptoms of anxiety (subscale with 12 items, score range 0-24) and depression (subscale with 6 items, score range 0-12). Higher scores refer to more symptoms.

#### Costs

The Medical Consumption Questionnaire (*i*MCQ) will be used to measure health care utilization. The *i*MCQ is a 31-item Dutch self-report questionnaire aimed to assess the direct costs of health care [17]. These are the costs of treatment, care and rehabilitation related to illness or injury and include expenditures for physicians and other health care professionals, care in hospitals and other institutions, and medication. We added extra items to the *i*MCQ to measure costs associated with contacts with a GP-MHW.

The Productivity Costs Questionnaire (*i*PCQ) will be used to assess productivity loss. The *i*PCQ is a 12-item Dutch self-report questionnaire aimed to measure indirect costs related to illness or injury [18]. These are the costs of productivity loss as a result of absence from work or inefficiency during paid or unpaid work.

#### Patient Acceptability

A Dutch translation of the Client Satisfaction Questionnaire-8 (CSQ-8) will be used to assess patient satisfaction with the study intervention [19]. The internal consistency of this scale in the Dutch population is very high. The 8-item self-report questionnaire has a score range from 8 to 32.

### Other Variables

Demographic information (eg, age, sex, educational level, and marital status), internet experience, and type and severity of main presenting symptom will be assessed at baseline. As a mediator, self-efficacy will be assessed by the Self-Efficacy Scale [20].

In addition, the GP and GP**-**MHW will be asked to fill out a number of questionnaires. The PCP’s attitude toward MUS will be assessed using a 24-item questionnaire. Potential determinants for health care professional implementation behavior will be examined using a selection of 13 items from the Determinants of Implementation Behavior Questionnaire [21]. The PCP’s attitudes with regard to risks and benefits of electronic health (eHealth) and their own computer skills will be assessed with the Dutch 18-item eHealth Attitude Questionnaire [22]. To assess PCP acceptability of Grip self-help, PCPs in the intervention group will complete the core item set of the CSQ-8, adjusted for use by health care professionals (CSQ-3).

#### Sample Size

Our power analysis is based on the effect estimates, calculated in our previous meta-analysis on the effectiveness of self-help interventions for MUS [11]. For HRQoL, we observed an effect size (Hedges g) of 0.66. As there was some evidence of publication bias toward larger effect sizes and because this meta-analysis also included studies with a waiting list control group, we based our calculations on an effect size of 0.5 (moderate effect). Without correcting for clustering by practice, the sample size based on an unpaired *t* test, given an effect size of 0.5, adopting power (1−beta) of .8, and alpha .05 2-sided, is 128. Accounting for 20% dropout, the number of patients that needs to be included is 1.25×128=160. On the basis of previous Dutch studies on MUS in general practice, we expect that a GP can include 4 patients during the inclusion period. To adjust the sample size for clustering by GP we calculated the design factor as: 1+(cluster size−1)×intraclass correlation coefficient (ICC). ICCs of 0.01 are recommended for the primary care setting [23], and the design factor then is 1+(4−1)×0.01=1.03. Consequently, a total of 1.03×160=165 patients need to be included, with an estimated number of 41 GPs.

### Statistical Analyses

Primary analysis will be performed on an intention-to-treat basis, meaning that all subjects that were allocated to either the intervention or the control group are included in the analysis and analyzed in the groups to which they were randomized. Secondary analyses will be performed on a per-protocol basis. The Grip self-help intervention is considered per-protocol if the last exercise has been completed. If, despite randomization, important baseline differences exist in prognostically important variables, they will be adjusted for by including them as covariates.

Differences in the effectiveness of Grip self-help compared with CAU will be analyzed using linear mixed-models (LMM), with HRQoL (RAND-36) and symptom severity (4DSQ) as outcomes. LMM allow correcting for dependence of (repeated) observations within patients as well as possible variations between practices. LMM have shown to be superior for the analysis of longitudinally correlated data and can optimally deal with missing values (no imputation needed) and cluster effects [24].

For the remaining analyses, missing values will be imputed using multiple imputation (MI). Both LMM with incomplete data and MI require the assumption of data being missing at random. Although this assumption is not testable, we will study the missing data mechanism by studying predictors of *missingness* of data using multivariable logistic regression analyses. The final imputation model will comprise all variables used in the analyses and all variables that predict *missingness* of a certain variable or its value.

The cost-effectiveness of Grip self-help compared with CAU will be investigated from a societal perspective, which includes costs in- and outside the health care sector (*i*MCQ and *i*PCQ). Results will be expressed in terms of incremental costs per quality-adjusted life year gained.

Acceptability of the Grip self-help intervention for patients and PCPs will be assessed using CSQ-8 and CSQ-3 scores.

To investigate which patient characteristics predict effectiveness of Grip self-help, Least Absolute Shrinkage and Selection Operator linear regression will be performed in the intervention group. Interaction terms of demographic variables and problem profile scores x treatment group will be entered as predictors, the physical component score of the RAND-36 at the end of treatment will be the outcome. For these analyses, the MI procedure will be performed separately in the treatment groups to allow for different associations between predictor and outcome in the Grip self-help and control condition.

To investigate which characteristics of PCPs predict effectiveness of Grip self-help, analyses of subgroups of these characteristics (eg, attitude toward MUS, eHealth attitude, and determinants for implementation behavior) will be performed, followed by statistical significance testing of the pertaining subgroup indicator xGrip self-help interaction term. As our sample calculation did not reckon with subgroup analyses, we consider these analyses exploratory in nature.

To investigate whether increased self-efficacy mediates treatment outcomes, we will use the regression-based method proposed by Preacher and Hayes [25].

## Results

Inclusion of PCPs started in December 2018, and enrolment is ongoing. The first results are expected to be submitted for publication in December 2021. Results will be reported according to the eHealth extension of the Consolidated Standards of Reporting Trials statement [26].

## Discussion

### Challenges

This paper presents the design of an RCT assessing the effectiveness and cost-effectiveness of Grip self-help: a personalized, Web-based, guided self-help intervention for patients with mild to moderate MUS in primary care.

Conducting this trial will involve several operational challenges. The first challenge is the recruitment of an adequate number of PCPs and participants. As an incentive, GPs are given €50 per included patient. However, as patients are selected by their GP based on past visits, there is a chance that patients are not experiencing current symptoms or difficulties and, therefore, are not motivated to participate in the study. Second, there is a chance of dropout in the control group, as these patients will not gain immediate access to the study intervention. This might lead to a lack of motivation to take part in follow-up assessments. To account for this challenge, patients in the control group will be offered access to the Grip self-help intervention after completion of the study. The last challenge is the potential nonusage of the Grip intervention. Previous studies have shown that nonadherence is a common problem in Web-based interventions [27]. To prevent nonusage, we have taken several measures. Patients will receive reminders when they have not logged into the Web-based platform. Also, the platform includes daily inspirational quotes and blogs to encourage daily use. In addition, log data enable us to track the amount of time patients spend using the intervention. Finally, guidance by the GP-MHW is offered throughout the intervention to motivate patients, answer questions, and overcome difficulties.

### Strengths and Limitations

Apart from these challenges, there are several strengths and limitations to the study. First, the Grip self-help intervention has a number of important strengths. As the intervention is provided in general practice, the intervention is easily accessible to a large group of patients. We hereby hope to also reach patients, who might not be willing to visit a mental health care facility to receive treatment. Also, the intervention is easy to implement in general practice because it is coherent with the current ways of working of PCPs. Strengths with regard to the study design are the follow-up period of 1 year, which allows for studying long-term effectiveness. Also, randomizing practices instead of patients will prevent contamination effects.

Of course, there are also a number of limitations to this study. First, self-selection of PCPs participating in the study may lead to selection bias, with an overrepresentation of PCPs having a special interest in either MUS or eHealth interventions. Second, the selection of patients by GPs also potentially causes selection bias. However, randomization takes place after the selection of patients, which limits this potential form of bias. Third, because of the nature of the intervention, patients, PCPs, and researchers will not be blinded to the study condition. This may lead to bias. Finally, outcome measures will be assessed using Web-based questionnaires. Although nearly all of the selected instruments were validated, traditional paper-and-pencil questionnaires were used in validation studies. This is of concern because psychometric properties might differ between different types of administration. However, several reviews have shown that Web-based testing usually produces very similar results compared with traditional testing [28,29].

### Conclusions

To our knowledge, this is the first study to combine the concepts of eHealth, self-help, and personalized medicine in the treatment of MUS. By improving the quality of life and reducing symptoms of patients with MUS, the Grip self-help intervention has the potential to reduce costs and conserve scarce health care resources.

## Appendix

Multimedia Appendix 1 Peer review report from funding body 1.

Multimedia Appendix 2 Peer review report from funding body 2.

Multimedia Appendix 3 Peer review report from funding body 3.

## Abbreviations

4DSQ: 4-Dimensional Symptom Questionnaire
CAU: care-as-usual
CSQ-8: Client Satisfaction Questionnaire-8
eHealth: electronic health
GP: general practitioner
GP-MHW: general practice mental health worker
HRQoL: health-related quality of life
ICC: intraclass correlation coefficient
iMCQ: Medical Consumption Questionnaire
iPCQ: Productivity Costs Questionnaire
LMM: linear mixed-models
MI: multiple imputation
MUS: medically unexplained symptoms
PCP: primary care professional
RAND-36: 36-item general health survey
RCT: randomized controlled trial
