# Supplementary material for: Personalized, Web-Based, Guided Self-Help for Patients With Medically Unexplained Symptoms in Primary Care: Protocol for a Randomized Controlled Trial
Source: JMIR Res Protoc. 2019 Oct 8;8(10):e13738. doi: 10.2196/13738 (PMC6913687; doi:10.2196/13738)
Supplement: Multimedia Appendix 1 [file resprot_v8i10e13738_app1.pdf]

Subsidieprogramma / Subsidy programme	:	**OnderzoeksProgramma GGz**
Dossiernummer / Dossier number	:	**60-63600-98-342**
Aanvrager / applicant	:	**Prof. dr. J.G.M. Rosmalen**
Projecttitel / Project title	:	**Master Your symptoms: personalized online Self-help for somatic symptom disorder (MYSelf)**
Beoordelingscode / Assessment code	:	**B.2017.00ACC**

1. Criteria

Legenda: G (Good), S (Sufficient), M (Moderate), U (Unsatisfactory)

1.1 Objective(s) and research question(s)

G	S	M	U
X			

Consider the following factors:

- The objective is clear and specific.
- The research question is clear, verifiable and connects with the objective.
- The scope and originality of the research question(s) is clearly described.
- There is a clear hypothesis.

The applicants present an interesting grant application with the aim to evaluate an online intervention targeting patients with mild to moderate somatic symptom disorder (SSD) in a pragmatic cluster randomized design. The applicants aim to investigate a subject of high clinical relevance. SSD are a burden for sufferers, they are also difficult to treat for doctors and psychotherapists and very costly for Society. The provided theoretical background and cited literature is consistent and includes all relevant current literature on SSD. The objective of the study is clearly described.
The research questions are original and clearly described.
The hypotheses with regard to effectiveness, feasibility, and cost-effectiveness are all clearly described. The research project represents an innovative personalised approach to deal with SSD in a primary care setting.

1.2 Strategy

G	S	M	U
X			

Consider the following factors:

- The research strategy is clear and connects to the research question.
- There is attention for gender differences, cultural background, age and the client perspective.
- An implementation strategy is added to the proposal:
  - The implementation strategy clearly describes how the project results will be distributed and secured.
  - The implementation strategy describes implementation activities.
  - It is clear how stakeholders will be involved to encourage implementation.

Research design

- There is a clear description of the research design.
- The research design is valid and connects to the research question.

Outcomes

- There is a clear description of the expected outcomes and intended results in terms of quality of care, quality of life, and satisfaction of patients. A study will be conducted to measure these outcomes and results.

Overall, the applicants present a well elaborated study design and methodology. They choose health related quality of life (measured by a well validated questionnaire) as their main outcome. All other outcomes are well described. One strength of the study is the innovative application of a personalized approach. Another strength of the study is the consideration of an elaborated cost-effectiveness analysis. In regular, online intervention studies suffer from high drop-out rates. The authors use a blended approach (with a GP mental health worker) who contacts and motivates participants at the beginning and every four weeks after the beginning of the intervention. I am absolutely convinced by the selection of the control condition, which is care as usual according to the dutch guidelines that are well presented in the grant application. In Addition, I would recommend to include a clear strategy of reminding patients to complete the intervention, if they do not fully adhere. This could be by e-mail, sms or telephone.
The applicants include a carefully arranged implementation strategy. Stakeholders as well as patients were involved in the design of the intervention. I am impressed by the elaborated design, that includes qualitative and quantitative methods.

1.3 Feasability

G	S	M	U
X			

Consider the following factors:

- It is possible to achieve the objective(s) using this strategy, duration and budget.
- The inclusion of participants in the research project is feasible using this strategy, duration and budget.
- Facilities/staff are available.
- The timetable is clear and realistic.

The reported fact that the intervention was created by well experienced researchers in the field of SSD in close collaboration with patients and health care professionals convinces me to assume a good feasibility of the study. Concept and milestones are clear and realistic. Recruitment of practices will be performed through healthcare Groups associated with a large Organization. In Addition electronic health records will be used to identify frequent attenders to facilitate recruitment of patients. The recruitment strategy represents a very promising Approach.

1.4 Project group

G	S	M	U
X			

Consider the following factors:

- Relevant expertise is available.
- Clients/ client representatives and/or their families are represented in the project group.
- A research organisation and healthcare institution are represented in the project group.
- All partners of the collaboration are represented in the project group.

All applicants are well experienced researchers in their individual fields and have excellent experience in interdiscipliary working fields. The main applicant is an expert of high renown in the field of SSD.
The prior activities of the applicants underline their ability to successfully perform the described Project in a good and funcioning cooperation. The president of the Pain Alliance Europe (PAE) as a representative of patients is member of the Project team. Therefore, clients are well represented in the project group.

1.5 Budget

G	S	M	U
X			

- There is a clear explanation of the requested budget.
- The requested budget is suitable for the grant application.
- If applicable: there is a clear explanation of the cofinancing.

The applicants aim to perform a large study in a Primary care setting, which is highly relevant, but also complex. Therefore, the requested budget of the study is relatively high. In my opinion, this is a down-to-earth estimation and underlines the high quality of this proposal. From my point of view, the budget is absolutely suitable for the grant application. The quality of such highly relevant research projects often suffer from inadequate financing (underfunding).

1.6 Overall quality assessment

G	S	M	U
X			

The present Research Grant application was conducted by an excellent and highly experienced Project Team. I am impressed by the preliminary work that led to the design of the Intervention. In my opinion this is an application dealing with a highly relevant Problem in an innovative manner. The scientific quality including design, recruitment strategy, and Intervention strategy seems excellent.
As a reviewer i recommend the funding of the Grant application with high priority.
