# Supplementary material for: Personalized, Web-Based, Guided Self-Help for Patients With Medically Unexplained Symptoms in Primary Care: Protocol for a Randomized Controlled Trial
Source: JMIR Res Protoc. 2019 Oct 8;8(10):e13738. doi: 10.2196/13738 (PMC6913687; doi:10.2196/13738)
Supplement: Multimedia Appendix 2 [file resprot_v8i10e13738_app2.pdf]

Subsidieprogramma / Subsidy programme	:	**OnderzoeksProgramma GGz**
Dossiernummer / Dossier number	:	**60-63600-98-342**
Aanvrager / applicant	:	**Prof. dr. J.G.M. Rosmalen**
Projecttitel / Project title	:	**Master Your symptoms: personalized online Self-help for somatic symptom disorder (MYSelf)**
Beoordelingscode / Assessment code	:	**B.2017.00ACD**

1. Criteria

Legenda: G (Good), S (Sufficient), M (Moderate), U (Unsatisfactory)

1.1 Objective(s) and research question(s)

G	S	M	U
X			

Consider the following factors:

- The objective is clear and specific.
- The research question is clear, verifiable and connects with the objective.
- The scope and originality of the research question(s) is clearly described.
- There is a clear hypothesis.

The scope and originality of research questions is well-described. The six research questions are clearly articulated and there are testable hypotheses proposed that are clinically relevant. The research questions are well-aligned/connected with the study objectives.
It is important to emphasize the clinical importance of the problem being studied and the innovative approach that is being tested. Somatic symptom disorder (SSD) is the new classification in DSM-V what was somatoform disorder in DSM-IV. It accounts for substantial disability as well as excess health care use. SSD is a prevalent and undertreated condition at the interface of general practice (GP) and mental health (MH). Many patients may not wish to access MH, and latter reserved for severe SSD. Innovative features of this study is the use of on-line tailored self-help strategies guided by a general practice mental health worker (GP MHW) who does have face-to-face contact at 4-week intervals with the patient. Also, while there are a variety of effective treatment strategies for SSD, including education, cognitive-behavioral therapy, mindfulness, and acceptance and commitment therapy, different strategies are more acceptable and effective for different types of patients. The proposed guided self-help intervention (MYSelf) allows personalization and tailoring of the treatment to the specific patient. The active involvement of a consortium of European organizations representing more than 300,000 patients with feedback on the intervention promises to make the ultimate self-help program more patient-centered which is especially important for SSD.

1.2 Strategy

G	S	M	U
X			

Consider the following factors:

- The research strategy is clear and connects to the research question.
- There is attention for gender differences, cultural background, age and the client perspective.
- An implementation strategy is added to the proposal:
    - The implementation strategy clearly describes how the project results will be distributed and secured.
    - The implementation strategy describes implementation activities.
    - It is clear how stakeholders will be involved to encourage implementation.

Research design

- There is a clear description of the research design.
- The research design is valid and connects to the research question.

Outcomes

- There is a clear description of the expected outcomes and intended results in terms of quality of care, quality of life, and satisfaction of patients. A study will be conducted to measure these outcomes and results.

The MYSelf trial is clearly described and tightly connected to the research questions. Strengths include situating the trial in real-world GP practices (that is, the pragmatic design), a care-as-usual control group, and randomization at the practice level to minimize contamination. The latter is especially important since both the GP and GP-MHW will be trained in MYSelf in the intervention group, and there training could spill over to control patients if randomization were to occur at the patient level.

There is a diverse sample of patients (increasing the generalizability of the results) and the authors will explore

patient factors that might modify effectiveness of the intervention. Inclusion and exclusion criteria are well-described and appropriate. The primary outcomes are patient-centered (physical function and, secondarily, symptoms and patient satisfaction), but other important outcomes such as acceptability of the intervention to doctors and patients, modifiers and mediators, and cost will also be evaluated.

The sample size estimates are well-described and appropriate and the hypothesized effect size of 0.5 is based on a meta-analysis and is reasonable. The analysis using repeated measures linear mixed models is state-of the art for clinical trials with longitudinal outcome assessments. Qualitative interviews with patients, GPs and GP-MHWs are proposed and should elucidate acceptability of MYSelf. Also a very rigorous cost analysis is described in detail.

The intervention is comprehensive, elegant, and nicely-tailored to the patient. The study team has done extensive work in developing the intervention drawing upon many clinicians, experts, and patients. Although many self-help exercises have been developed, a sophisticated algorithm allows patients to select from a menu most relevant to their symptoms and preferences. Success of the self-help program will be accentuated by facilitation from the GP-MHW who could provide additional guidance and motivation. Finally, a strong implementation plan is described.

1.3 Feasability

G	S	M	U
X			

Consider the following factors:
- It is possible to achieve the objective(s) using this strategy, duration and budget.
- The inclusion of participants in the research project is feasible using this strategy, duration and budget.
- Facilities/staff are available.
- The timetable is clear and realistic.

The trial design is described clearly, scientific valid, and well-connected to the research questions. The outcomes being assessed are valid, well-described, and appropriate to the intervention being tested. The large recruitment network of potential GPs assures the likelihood of meeting enrollment targets. The goal of recruiting 4 SSD patients from each participating GP is a reasonable number and feasible to achieve. The fact that both GP referral of potential patients as well as identification of patients through electronic health records further assures meeting enrollment targets. The study timeline is clear and realistic. The facilities and staff for this trial are excellent.

1.4 Project group

G	S	M	U
X			

Consider the following factors:
- Relevant expertise is available.
- Clients/ client representatives and/or their families are represented in the project group.
- A research organisation and healthcare institution are represented in the project group.
- All partners of the collaboration are represented in the project group.

This is one of the strongest teams not only in Europe but internationally to conduct a treatment trial of SSD. Their preliminary work is impressive and sets the stage nicely for this trial. Experts from several universities are involved. The study team's ongoing engagement with general practice not only enhances the conduct of this trial but also paves the way for subsequent implementation of their findings into real-world clinical practice. The partnership with patient organizations representing large numbers of stakeholders informs the intervention and also increases the likelihood of advocacy for uptake into practice and conversations with leaders who develop policies and practice guidelines. Since the focus is on web-based self-help strategies, the involvement of patient stakeholders might also independently benefit individuals with SSD outside the health care system.

1.5 Budget

G	S	M	U
X			

- There is a clear explanation of the requested budget.
- The requested budget is suitable for the grant application.
- If applicable: there is a clear explanation of the cofinancing.

The budget is nicely detailed and suitable for the grant application.

1.6 Overall quality assessment

G	S	M	U
X			

This is a very important well-designed trial on a condition (SSD) very prevalent in clinical practice, disabling, undertreated, and costly to the health care system and society. The use of personalized self-health strategies done on-line with guidance from a mental health worker is innovative and feasible to implement if effective. The active involvement of patients as well as GPs as important stakeholders further enhances the subsequent impact of the intervention. All parts of the application are well-developed. The study team is one of the strongest

international group of investigators in this condition and thus well-positioned to both complete this trial, disseminate its findings, and promulgate eventual uptake into real-world practice.
