# Supplementary material for: Personalized, Web-Based, Guided Self-Help for Patients With Medically Unexplained Symptoms in Primary Care: Protocol for a Randomized Controlled Trial
Source: JMIR Res Protoc. 2019 Oct 8;8(10):e13738. doi: 10.2196/13738 (PMC6913687; doi:10.2196/13738)
Supplement: Multimedia Appendix 3 [file resprot_v8i10e13738_app3.pdf]

Subsidieprogramma / Subsidy programme	:	**OnderzoeksProgramma GGz**
Dossiernummer / Dossier number	:	**60-63600-98-342**
Aanvrager / applicant	:	**Prof. dr. J.G.M. Rosmalen**
Projecttitel / Project title	:	**Master Your symptoms: personalized online Self-help for somatic symptom disorder (MYSelf)**
Beoordelingscode / Assessment code	:	**B.2017.00D56**

1. Criteria

Legenda: G (Good), S (Sufficient), M (Moderate), U (Unsatisfactory)

1.1 Objective(s) and research question(s)

G	S	M	U
X			

Consider the following factors:

- The objective is clear and specific.
- The research question is clear, verifiable and connects with the objective.
- The scope and originality of the research question(s) is clearly described.
- There is a clear hypothesis.

This is quite a large and complex study with many components. There were some aspects with which I was not familiar so please read this review with that in mind. I thought the study was well conceptualised with a clear rationale. SSD is a poorly managed disorder in my experience, and consumes a lot of health care resources because clinicians lack skills in responding to it in a helpful way. Consequently a lot of people with SSD experience prolonged impaired functioning, dissatisfaction with health services, and frequently futile medical and surgical interventions. This study is therefore very timely. It is certainly a very original study, and a large and robust study for a pilot project. There are clear hypotheses for the study, and clear primary and secondary outcomes which connect strongly with the research questions. There is a good range of standardised outcome measures.

1.2 Strategy

G	S	M	U
X			

Consider the following factors:

- The research strategy is clear and connects to the research question.
- There is attention for gender differences, cultural background, age and the client perspective.
- An implementation strategy is added to the proposal:
  - The implementation strategy clearly describes how the project results will be distributed and secured.
  - The implementation strategy describes implementation activities.
  - It is clear how stakeholders will be involved to encourage implementation.

Research design

- There is a clear description of the research design.
- The research design is valid and connects to the research question.

Outcomes

- There is a clear description of the expected outcomes and intended results in terms of quality of care, quality of life, and satisfaction of patients. A study will be conducted to measure these outcomes and results.

There is a very strong research design and a good outline of the basis for the study in previous work. The research strategy is clearly outlined and strongly linked to the research question. The recruitment and data collection processes appear robust and there is a clear plan of implementation. There is good evidence of stakeholder engagement. The outcome measures are all appropriate and meaningful. There is also a good range of clinical social and health service utilisation.

1.3 Feasability

G	S	M	U
X			

Consider the following factors:

- It is possible to achieve the objective(s) using this strategy, duration and budget.
- The inclusion of participants in the research project is feasible using this strategy, duration and budget.
- Facilities/staff are available.
- The timetable is clear and realistic.

The project appears to be feasible, and the time frame seems appropriate. The process of recruitment is sound and will meet the objective of preventing contamination. The research team is very experienced and has a very wide range of skills and experience. I can't comment on the budget.

1.4 Project group

G	S	M	U
X			

Consider the following factors:

- Relevant expertise is available.
- Clients/ client representatives and/or their families are represented in the project group.
- A research organisation and healthcare institution are represented in the project group.
- All partners of the collaboration are represented in the project group.

Yes, a very high level of research expertise. Good client involvement through the members of the research team. Mr van Griensven brings a strong consumer perspective. The academic affiliations of the research team show it to be strongly linked to academic institutions and there is also good clinical skills in the research team.

1.5 Budget

G	S	M	U
X			

- There is a clear explanation of the requested budget.
- The requested budget is suitable for the grant application.
- If applicable: there is a clear explanation of the cofinancing.

I feel unable to comment on the budget

1.6 Overall quality assessment

G	S	M	U
X			

Overall I thought this was a very impressive study. The MYSelf intervention looks very promising; it is something I would like to see more widely applied. The proposal is well developed and clearly described.
